# Meaningful Social Inclusion and Mental Well-Being Among Autistic Adolescents and Emerging Adults: Protocol for a Community-Based Mixed Methods Study

**DOI:** 10.2196/52658

**Published:** 2024-03-14

**Authors:** Darcy Jones McMaughan, Casey Lewis, Amy McGehee, Dani Noreen, Elliot Parker, Michael M Criss

**Affiliations:** 1 Human Development and Family Science Oklahoma State University Stillwater, OK United States; 2 Neurodiversity Unbound Aurora, CO United States

**Keywords:** autism, community-based, mixed methods, social inclusion, well-being

## Abstract

**Background:**

In the United States, autistic people face high rates of co-occurring mental illnesses and premature death due to self-harm, which are indicators of threats to mental well-being. Social inclusion may enhance mental well-being and resilience among autistic people. According to Simplican and colleague’s (2015) model of social inclusion for people with intellectual and developmental disabilities, social inclusion is an interaction between community participation and interpersonal relationships. There is limited research on social inclusion that includes the integration of interpersonal relationships and community participation among autistic people or the impact of social inclusion on the well-being of autistic people. Additionally, little evidence exists regarding how autistic people prefer to be included in the community or form interpersonal relationships.

**Objective:**

The long-term objective of this project is to improve social inclusion factors to support the mental well-being of autistic people. This protocol describes a community-based, mixed methods pilot study to develop a definition of meaningful social inclusion for autistic people and to understand the relationship between meaningful social inclusion and mental well-being among autistic adolescents and emerging adults.

**Methods:**

The project uses a community-based, sequential mixed methods design with a formative phase (Phase 1) that informs a survey phase (Phase 2) and concludes with a process evaluation of the community engagement process (Phase 3). During Phase 1, we will recruit 10 community partners (autistic adults and stakeholders) and conduct sharing sessions to cocreate a definition of meaningful social inclusion and a survey of meaningful social inclusion and well-being. During Phase 2, we will recruit 200 participants (100 autistic adolescents and emerging adults and 100 caregivers) to complete the survey. We will examine whether meaningful social inclusion predicts well-being given sociodemographic factors using ordered logistic regression, with well-being categorized as low, medium, and high. During Phase 3, the community partners from Phase 1 will complete a survey on their experiences with the project.

**Results:**

Ethics approval was obtained for this project in March 2023. We have recruited community partners and started the Phase 1 focus groups as of September 2023. Phase 2 and Phase 3 have not yet started. We expect to complete this study by March 2025.

**Conclusions:**

Using a community-based, mixed methods approach, we intended to develop a definition of meaningful social inclusion for autistic people and understand the role meaningful social inclusion plays in the well-being of autistic people.

**International Registered Report Identifier (IRRID):**

PRR1-10.2196/52658

## Introduction

### Overview

Mental well-being is a holistic conceptualization of mental health characterized by a lack of symptoms of mental conditions and the presence of positive feelings [1]. Compared to nonautistic people, autistic people often experience poor mental well-being [2], reduced quality of life [3], higher rates of suicidality and premature death due to suicide [4-6], and greater rates of mental health conditions [7-9]. Worldwide, across decades, and across age groups, autistic people have a twofold increase in mental health issues compared to people without autism. For example, rates of mental health issues among the general population in the United States hit an all-time high during the COVID-19 pandemic, with an estimated 40% reporting mental health challenges [10], while rates of co-occurring mental health issues among autistic adolescents and emerging adults during a “typical” time period are as high as 82% [11]. The total size of the autistic population is unknown due to women, gender-diverse people, and people of color being historically underdiagnosed and adult-diagnosed autistic people not being captured in prevalence rates. Even so, current estimates suggest that 1 in 44 children in the United States are diagnosed with autism [12]. The impact of poor mental well-being among autistic people impacts a substantial proportion of the US youth population. The burden of poor mental well-being in the autistic community increases with age [13,14] and shortens life expectancy [5].

A complex interplay of internal and external factors may explain disparities in mental well-being between autistic people and nonautistic people. While endogenous factors such as genetics and neurology play a role, systemic, compounding stressors exogenous to the autistic person can also influence mental well-being. These modifiable, external factors include experiences such as being bullied, masking, and feeling accepted. Autistic people experience higher rates of bullying compared to nonautistic people, and these experiences are linked to a greater risk of mental health issues [15]. Masking (also known as camouflaging) describes the efforts of autistic people to hide autistic traits and avoid negative experiences such as bullying. Similar to bullying, masking is also associated with poorer mental well-being [16-19]. Conversely, when autistic people feel accepted, they report better mental well-being. Higher levels of acceptance by nonautistic people are associated with lower levels of mental distress [20]. Contrary to stereotypes of autistic people, research confirms that interpersonal relationships are important to the autistic well-being [20], and higher levels of community participation are associated with better mental well-being [21,22].

However, current research suggests that autistic people face individual-level social inclusion barriers [20,23] and are less likely to participate in the community [24] or form interpersonal relationships [25] compared to nonautistic people. Additionally, this literature primarily focuses on how autistic people should change to adapt to communities and other people, rather than on how communities and other people could adapt to include autistic people [26]. Because they do not center on the experiences of autistic people, many of these studies focus on independence rather than autonomy and conformity to neurotypical standards as criteria for social inclusion and enforced normalization. Given the historic emphasis on children [7], it is imperative to focus on the mental well-being of autistic adolescents and emerging adults. Social inclusion may hold the key to implementing sweeping changes to improve mental well-being for autistic adolescents and emerging adults [23,27], particularly as identification with social groups is associated with higher levels of mental well-being among autistic people [1]. Social inclusion and mental well-being are research priorities for the autistic community [28], as is shifting the focus from “fixing” the individual to addressing systemic issues external to the autistic person [29].

To the best of our knowledge, no study has integrated both aspects of social inclusion, included the voices of autistic people, and focused on mental well-being. These 2 relationship contexts (interpersonal relationships and community participation) are instrumental in promoting adaptive mental and physical health among autistic people. Interpersonal relationships are important to the well-being of autistic people [20], and higher levels of community participation are associated with better mental well-being [21,22]. This project fills a critical need to develop an understanding of meaningful social inclusion that includes both interpersonal relationships and community participation for autistic people and use this to understand the impact of social inclusion on mental well-being. Without an understanding of how social inclusion factors shape mental well-being, we cannot identify and implement effective and efficient community-level change to improve the well-being and quality of life of autistic people. In the absence of this knowledge, rates of co-occurring mental health conditions, the resulting diminished quality of life, and premature deaths will remain high. Filling this gap with this study is critical, as this information is necessary for policy and planning to improve mental health parity among autistic people.

### Conceptual Framework

There is no agreed-upon conceptual framework for social inclusion. However, the ecological model of social inclusion for people with intellectual and developmental disabilities by Simplican and colleagues [30] provides a road map for understanding, measuring, and modifying social inclusion. According to this model, social inclusion is defined as the interaction of interpersonal relationships (family, staff, friends, acquaintances, and partners) and community participation (leisure activities, political and civic activities, employment and education, access to goods and services, and religious and cultural activities).

### Study Aims

The long-term objective of this project is to improve social inclusion factors to support the mental well-being of people with autism. Our goal is to address the problem of high rates of poor mental well-being by understanding how social inclusion is associated with this phenomenon. We focus on adolescence and emerging adulthood because this is a key developmental period for autistic people and a challenging time for mental well-being [31,32]. We will meet our goal through two specific aims: Aim 1: develop a working definition of meaningful social inclusion for autistic people living in the community that is cocreated with the autistic community; and Aim 2: delineate variation in mental well-being by social inclusion (interpersonal relationships and community participation) among autistic adolescents and emerging adults. Our hypothesis is that higher levels of social inclusion will be associated with higher rates of mental well-being.

## Methods

All protocols and materials will follow the Academic Autism Spectrum Partnership in Research and Education practice-based guidelines for research coproduced with autistic people [33].

### Study Design

#### Overview

The project uses a community-based, sequential mixed methods design with a primary formative phase that will inform a subsequent secondary survey phase [34] and a tertiary phase that consists of a process evaluation of the community engagement process. This design has been used successfully to capture community member experiences and influence policy changes [35]. In Phase 1, we will conduct sharing sessions with members of the autistic community to cocreate (1) a working definition of meaningful social inclusion and (2) an adaptation of a survey of social inclusion and well-being. In Phase 2, we will deploy the cocreated survey in the autistic community to gather data on their level of social inclusion and mental well-being. In Phase 3, we will evaluate the research processes. Findings from each phase will be integrated using triangulation with the autistic community. We will consider where findings from each part agree (convergence), result in complementary information (complementarity), or disagree (discrepancy).

#### Phase 1: Formative Research

Sharing sessions will be held virtually through Zoom (Zoom Video Communications), recorded, and transcribed for analysis. Sharing sessions will be facilitated through a series of open-ended questions developed to capture the lived experiences of meaningful social inclusion of autistic community members. Community members participating in the sharing sessions will be provided with informed consent, a 1-page description of the study, the open-ended questions, the draft survey, and the background of the research team. Multiple meetings will be scheduled to (1) develop an understanding of meaningful social inclusion, (2) select and refine survey items, (3) review analysis results, and (4) determine the best ways to disseminate the results that align with the community’s agenda. Over the course of the project, the timing, number, and format of the meetings will be negotiated with community partners [36].

#### Phase 2: Adapted Survey of Social Inclusion and Mental Well-Being

This aspect of the project follows a cross-sectional survey design in which data are captured at a single point in time. Autistic adults living in the community will report on social inclusion and mental well-being using adaptations of existing survey instruments created with community members during the sharing sessions. Data will be gathered through an anonymous web-based questionnaire hosted through the Qualtrics (Silver Lake) platform.

#### Phase 3: Process Evaluation

Satisfaction with community engagement will be measured by asking community partners at the end of the study to report on their experiences with the community engagement process.

### Measures

#### Overview

Our main variables of interest are meaningful social inclusion and mental well-being. We will measure the identity of autistic people and address potentially confounding variables through sociodemographic variables associated with social inclusion and mental well-being. Whenever possible, short-form versions of instruments will be used to reduce the burden on participants. Items will also be adapted for completion by caregivers reporting on autistic adolescents and emerging adults in their care. Caregivers will be asked if the autistic adolescents and emerging adults in their care also completed the survey to create a subgroup of caregivers providing proxy responses and caregivers providing additional responses.

#### Identity of Autistic People

The identity of autistic people will be measured using current guidelines for establishing rigor to generalize to autistic people [37], through self-identification, age at diagnosis, diagnosis status (self vs community), and the Ritvo Autism Asperger Diagnostic Scale-14 [38]. This measure will be used for autistic people who will complete the survey. Caregivers will be asked if they care for an autistic adolescent or emerging adult, whether the autistic adolescent or emerging adult was professionally diagnosed, the current age of the person they provide care for, and the age the person was diagnosed.

#### Social Inclusion

Social inclusion will be assessed using adaptations of the Temple University Community Participation (TUCP) measure [39] for community participation and the Friendship Questionnaire (FQ) for interpersonal relationships [40]. The TUCP contains 26 items that measure independent and self-directed community participation over the last 30 days in the following domains: community activities, employment, education, and volunteering. The TUCP asks (1) for the number of days in the past 30 days that the person engaged in a community activity (0-30); (2) if the activity is important to the person (1=yes or 0=no); and (3) if the activity was done enough, not enough, or too much (−1=not enough, 0=enough, or 1=too much). Although initially created for use with people with complex and chronic mental health conditions, the TUCP has been used to assess community participation among autistic adults [24,41,42].

The FQ contains 35 items that measure multiple aspects of interpersonal relationships, such as having best friends (“I have one or two particular best friends”) and the characteristics of friends (“In terms of interests, how similar to your friends do you tend to be?”). The FQ was designed for use with nonautistic and autistic adults [40].

#### Mental Well-Being

Mental well-being will be captured through the Warwick-Edinburgh Mental Well-being Scale (WEMWBS) [43] and the Depression Anxiety Stress Scales-21 (DASS-21) [44]. The WEMWBS is a 14-item questionnaire measuring positive mental well-being through positive emotions, positive mental health, and psychological functioning. Each item is a positively worded statement (eg, “I’ve been feeling good about myself”) with a 5-point Likert-type scale (1=none of the time to 5=all the time) indicating how often the statement applied to the person over the past 2 weeks. An overall score is calculated by totaling the scores for each item. Higher scores indicate higher levels of mental well-being. The WEMWBS has good content and construct validity, face validity, cross-cultural validity, test-retest reliability (intraclass correlation =.83), and internal consistency, with Cronbach α ranging from 0.89 to 0.93 [43,45]. The WEMWBS has been extensively used to measure mental well-being among autistic adults [1,46]. The DASS-21 contains 3 self-report scales measuring depression, anxiety, and stress. The DASS-21 has been found to function well as a measure of psychological distress among autistic people [47].

#### Sociodemographic Variables

Sociodemographic variables will include age, gender, race and ethnicity, rurality, income, education, employment status, co-occurring conditions, level of support need, and social functioning using the Social Functioning Questionnaire [48]. Gender, education, race and ethnicity, and co-occurring conditions are items derived from the National Survey on Health and Disability. Gender will include options for gender-diverse people. Employment status is based on items used in the 2021 Behavioral Risk Factor Surveillance System. Participants will be asked to estimate their household income and will be presented with multiple-choice income options. The level of support needed will be determined using items representing assistance needed for activities of daily living and instrumental activities of daily living.

Rurality will be identified using the self-reported residence zip code of the participant’s permanent residence and categorized based on Rural-Urban Commuter Area (RUCA) codes. RUCA codes classify US census tracts on a continuum of rural to urban categories using patterns of daily commuting, population density, and measures of urbanization. Whether a participant originated from a rural community will be determined based on residence in a zip code associated with large rural, small rural, or isolated RUCA codes.

#### Satisfaction With Community Engagement

Satisfaction with community engagement will be measured by asking community partners who participated in the sharing sessions to complete a Community Engagement Questionnaire [36] describing their experiences with the project: (1) What has been your experience with participating in this research study? (2) How satisfied were you with your contribution to this research study? (3) How could your experience be improved? and (4) Is there anything else you would like to add?

### Study Participants and Recruitment

Participants will be recruited on the web through groups such as the Autism Foundation of Oklahoma, Autistic Adults of Oklahoma, AutismStillwater’s website, AutismOKC’s website, tribal organizations, and state department health services caseworkers. Our community partners will leverage their community contacts in each of these groups for the recruitment of participants for the study.

The intended participants for Phase 1 are autistic adults and caregivers of autistic young people. The participants will be older than 18 years and reside in Oklahoma. For Phase 2, the intended participants are (1) autistic adolescents (aged between 10 and 18 years) and emerging adults (aged between 19 and 25 years) residing in Oklahoma, fluent in English, who have a community diagnosis of autism or meet the Ritvo Autism Asperger Diagnostic Scale-14 cutoff for autism and (2) caregivers of autistic adolescents and emerging adults. Phase 3 participants are the same participants from Phase 1. Exclusion criteria include communication impairment and an inability to consent.

For Phase 1 and Phase 3, we will recruit up to 10 autistic community partners to participate in the sharing sessions, and each partner will be paid US $400 for their participation. For Phase 2, we will recruit 100 autistic adolescents and emerging adults living in the community and 100 caregivers to complete the adapted survey. The most recent electronic survey deployed by the Autism Foundation of Oklahoma resulted in 270 respondents. Based on this, we believe 200 respondents is feasible. We will not incentivize the survey. Recent web-based surveys deployed by the research team that were incentivized (ie, had a gift card raffle) resulted in a high number of bots and scammers completing the survey for monetary gain. This, combined with the relatively high number of participants the Autism Foundation of Oklahoma had on their surveys who were not incentivized, led the research team to drop the use of incentives.

### Data Analysis

#### Phase 1: Formative Research and Phase 3: Process Evaluation

The data from the sharing sessions and community engagement questionnaire will be analyzed and interpreted using iterative inductive and deductive thematic analyses. Data will be developed through verbatim transcription of audio recordings, analyzed using Dedoose (SocioCultural Research Consultants), and transformed into concept maps. The transcripts and texts will be reviewed several times and coded based on themes to reduce and display the data and draw conclusions. This process involves (1) familiarizing the research team with the data, (2) coding the data, (3) using the codes to develop initial themes, (4) reviewing the themes as a team, (5) defining the themes, and (6) writing up the results. Inductive codes will be based on emerging codes from the data, and deductive codes will be based on the literature and the open-ended sharing session and process evaluation questions. The credibility of the outcomes will be verified with community members to help ensure we capture the experiences of the community members.

#### Phase 2: An Adapted Survey of Social Inclusion and Mental Well-Being

We will examine whether meaningful social inclusion predicts well-being given sociodemographic factors using ordered logistic regression, with well-being categorized as low, medium, and high [49]. Models will be adjusted using participant characteristics to hold influences outside of social inclusion constant.

#### Power Analysis

Qualitative data will be collected until saturation is reached. For quantitative analysis, logistic regression is a nonlinear model, and thus, probability at the mean and 1 SD above the mean is necessary for the power analysis. These values are not available in the literature. We will develop these probabilities through this pilot study for use in a larger study. However, in general, a sample size over 100 with cell counts over 10 is necessary for logistic regression.

### Ethical Considerations

Ethics approval for Phase 1 was obtained for this project in March 2023 from the Institutional Review Board of Oklahoma State University (IRB-23-46). Phase 2 and Phase 3 of this protocol will be reviewed and approved, and electronic informed consent or assent, where appropriate, will be attained before beginning any aspect of the study.

## Results

The study was funded by Oklahoma Center for the Advancement of Science and Technology in January 2023. Ethics approval was obtained for this project in March 2023. We have developed Phase 1 materials, recruited community partners, developed a draft of the survey, and started the Phase 1 focus groups as of September 2023. Phase 2 and Phase 3 have not started yet. We expect to complete this study by March 2025.

## Discussion

### Expected Outcomes and Potential Impact

The expected outcomes of this protocol include a definition of meaningful social inclusion for autistic people and an enhanced understanding of the role meaningful social inclusion plays in the well-being of autistic people. The research findings may generate new knowledge on meaningful social inclusion that addresses community-level needs and limited social inclusion among autistic adolescents and emerging adults. This project could advance our understanding of how to improve social inclusion, reduce poor mental well-being among autistic adolescents and emerging adults, and advance our scientific understanding of facilitating social inclusion and integration of autistic people in communities.

Results will be integrated into the Autism Foundation of Oklahoma’s training and awareness programs on, for example, employment, education, and criminal justice. The results will also be used to develop new training and awareness programs focused on social inclusion. The proposed study will provide preliminary data for developing interventions for testing (eg, modifying the Autism Foundation of Oklahoma’s current training program for employers to hire autistic people) and lay the groundwork for larger studies on enhancing quality of life and social inclusion factors such as interpersonal relationships and community participation for autistic adolescents and emerging adults.

### Potential Difficulties and Limitations

We assume that social inclusion is associated with better mental well-being. However, it could be that better mental well-being is associated with higher levels of social inclusion; that is, better mental well-being leads to more social inclusion. This is an issue of endogeneity—when the outcome variable is a predictor and not simply a response (simultaneity bias). We can test for this relationship using the Hausman test for endogeneity, and we also included variables that can be used as statistical instruments should we need to use an instrumental variable analysis to control for endogeneity.

### Strength of the Study

Most research on social inclusion and autistic people focuses on exclusion rather than inclusion. This research is typically conducted on autistic children in primary school settings and often excludes the lived experiences of autistic people. This can result in outcomes and recommendations that do not align with the experiences and goals of the autistic community. As such, several strengths of this study lie in our focus on social inclusion rather than exclusion, our centering of the experiences of autistic adolescents and emerging adults, and our use of a community-based mixed methods approach to engage the autistic community. By focusing on the lived experiences of autistic people, we increase the likelihood that the outcomes of this project will have a meaningful impact on the well-being of autistic people.

## Appendix

Multimedia Appendix 1 Peer review reports.

## Abbreviations

DASS-21: Depression Anxiety Stress Scales-21
FQ: Friendship Questionnaire
RUCA: Rural-Urban Commuter Area
TUCP: Temple University Community Participation
WEMWBS: Warwick-Edinburgh Mental Well-being Scale
