# Supplementary material for: Meaningful Social Inclusion and Mental Well-Being Among Autistic Adolescents and Emerging Adults: Protocol for a Community-Based Mixed Methods Study
Source: JMIR Res Protoc. 2024 Mar 14;13:e52658. doi: 10.2196/52658 (PMC10979331; doi:10.2196/52658)
Supplement: Multimedia Appendix 1 [file resprot_v13i1e52658_app1.pdf]

Review Form 1 (Subgrantee)

Project Lead: *DJ McMaughan*

Project Title: *Impact of social inclusion on mental wellbeing among autistic adolescents and emerging adults*

Organization: *Oklahoma State University*

Issue	Area	Description of Issue
	Human Subjects	
	Vertebrate Animals	
	Recombinant DNA	
	Radioisotopes	
	Narcotics and Dangerous Drugs	
	Biological Hazards	

Comments:

Evaluation Criteria

1. Significance

Strengths:

The current study addresses mental health concerns of autistic people. Rates of co-occurring mental health issues among autistic adolescents and emerging adults are as high as 82% (double that of people without autism). Current estimates suggest that 1 in 44 children in the U.S. are diagnosed with autism. The proposed study will provide important preliminary data for developing training and interventions focused on social inclusion. Results will be integrated into the Autism Foundation of Oklahoma's training and awareness programs.

Weaknesses:

None noted.

2. Approach

Strengths:

The propose to conduct sharing sessions to co-create and then deploy a survey of social inclusion using two samples of people from the autistic community: 10 community partners (autistic adults and stakeholders who will help create the survey) and 200 participants (100 autistic adolescents and emerging adults and 100 caregivers). In the first (qualitative) phase, they will conduct sharing sessions with members of the autistic community to co-create: 1) a working definition of social inclusion and 2) adaptation of a survey of social inclusion. In the second (quantitative) phase, they will deploy the survey in the autistic community to gather data on level of social inclusion and mental wellbeing. Finally, they will conduct a process evaluation of the research processes. Findings from each phase will be integrated using triangulation with the autistic community. Participants will be recruited online through the Autism Foundation of Oklahoma, Autistic Adults of Oklahoma, AutismStillwater.org, AutismOKC.org, tribal organizations, and state department health services caseworkers.
I appreciate their positionality statements.

Weaknesses:

I am curious about the inclusion of only one measure of well-being. Wouldn't it be valuable to include other specific measures (if validated in the target population), such as anxiety and depression, or even measures related to self-harm given the elevated rates in that population?

3. Innovation

Strengths:

The proposed study is one of fewcommunity-engaged projects with the autistic community, a best practice particularly when working with communities that have been marginalized. No study has integrated both aspects of social inclusion, included autistic voices, and focused on mental wellbeing. It also changes the focus from problem-fixing (autistic people need to change to fit into society) to possibility-creating (we can create a community inclusive of autistic people). Finally, most studies of autistic individuals focus on children.

Weaknesses:

They state that "no research exists that examines social inclusion and mental wellbeing" (pg 1), but they must mean that no research like this exists for an autistic population since a quick pubmed search results in over a thousand articles.

4. Investigators

Strengths:

The research team is uniquely suited to conduct this type of research. The PI (Dr. McMaughan) is a neurodivergent, disabled scholar of disability, an expert on systems improvement for disabled people. They are experienced in survey creation and data analysis, qualitative and mixed-methods research, and community engagement. They are a member of Autistic Adults of Oklahoma, and co-chair of the Autism Foundation of Oklahoma's Public Health and Safety Committee. They have recruited community participants for the development of the survey. The PI and collaborators have worked together in the past, including studies of psychiatric hospitalizations and autistic adolescents and emerging adults. All members of the team appear to have extensive personal and professional backgrounds in this topic. They will leverage their community contacts for recruitment.

Weaknesses:

None noted.

5. Environment

Strengths:

OSU-Stillwater has adequate resources to support the proposed project, including Qualtrics licenses and statistical software, as well as research support services.

Weaknesses:

None noted.

Response to Previous Reviewers (if applicable)

The authors revised the proposal substantially from the earlier submission. They addressed previous concerns adequately (whenever still relevant).

Overall Evaluation

The proposed mixed methods study addresses social inclusion and mental health among autistic adolescents and emerging adults. They propose to develop a new conceptualization of social inclusion that includes both interpersonal relationships and community participation for autistic people and then to assess the impact of social inclusion on the mental well-being. They will use sharing sessions to co-create a survey of social inclusion, and then will use the survey within two samples of people from the autistic community: 10 community partners (autistic adults and stakeholders who will help create the survey) and 200 participants (100 autistic adolescents and emerging adults and 100 caregivers). At the end, they will conduct a process evaluation of the research processes. Throughout, they will work closely with the autistic community. The study's innovation is that it will be the first to integrate two aspects of social inclusion into one measure, include autistic voices, and focuse on mental wellbeing. The research team is uniquely well-suited to conduct this type of research, with extensive lived experiences that complement their professional expertise in survey research methodology, qualitative methods, and community engagement. They also have numerous networking connections to facilitate recruitment. No major weaknesses were noted.

Review Form 2 (Subgrantee)

Project Lead: *DJ McMaughan*

Project Title: *Impact of social inclusion on mental wellbeing among autistic adolescents and emerging adults*

Organization: *Oklahoma State University*

Issue	Area	Description of Issue
	Human Subjects	
	Vertebrate Animals	
	Recombinant DNA	
	Radioisotopes	
	Narcotics and Dangerous Drugs	
	Biological Hazards	

Comments: *no concerns*

Evaluation Criteria

1. Significance

Strengths:

Autistic adolescents and young adults has high rates of mental illness and self-harm (including death). The proposal suggests that social inclusion is a factor in mental well-being in this population. The overall objective of this proposal is to define meaningful social inclusion and then to measure two domains of social inclusion and assess their association with mental health. Results could provide important preliminary data for newinterventions as well as incorporation into training programs. The long-term goal is to improve social inclusion factors and so improve mental well-being.

Weaknesses:

none noted

2. Approach

Strengths:

The research strategy is nicely written and sufficiently detailed.

The research methods are straightforward and measures are clearly defined.

Analysis plans are appropriate. with justified sample sizes.

The discussion of potential pitfalls is excellent.

The timeline appears feasible.

Weaknesses:

A more thorough discussion of previous research supporting the link between social inclusion and mental well-being would strengthen the premise of the study.

It is unclear howthe caregivers data will be used (Aim 2). Howmuch of these data independent of that provided by the 100 autistic individuals? Howdo the two data sources combine for analysis?

Information about the internal and external validity of the questionnaires to be used is not provided.

3. Innovation

Strengths:

The focus on adolescent and emerging adult autistic individuals is innovative; most research focuses on children.

The participation of the autistic community in defining social inclusion, and the extent of engagement with the autistic community, are innovative.

Focusing on social inclusion, and attempting an operational definition of it, is innovative.

Weaknesses:

none noted

4. Investigators

Strengths:

Dr. McMaughan will well-qualified to lead this study. She has the academic and research background necessary as well as the lived experience. She has conducted research in social isolation previously, as well as in mental health and with autistic children.

Dr. Criss brings relevant experience to the research team. He has the appropriate expertise to support and assist with this study.

Community partners and consultants have been identified.

Weaknesses:

none noted

5. Environment

Strengths:

Excellent resources and facilities

Weaknesses:

none noted

Response to Previous Reviewers (if applicable)

Overall Evaluation

This is an innovative proposal from a highly qualified PI and team that seeks to investigate the association between social inclusion and mental health in adolescent and emerging adult autistic individuals. The environment and resources are excellent and the team has excellent access to the target population. The first aim deals with generating a definition of social inclusion via focus groups. The second aim is a quantitative exploration of social isolation domains and mental health. The methods are appropriate and sample sizes appear reasonable. The major weakness is the underdeveloped argument for the link between social inclusion and mental health. The minor weaknesses can be addressed by clarification of the use of caregiver data and psychometric details of the surveys. This is overall a potentially impactful study that is well designed and highly feasible.

Review Form 3 (Subgrantee)

Project Lead:
Project Title:
Organization:

Issue	Description of Issue
Human Subjects	
Vertebrate Animals	
Recombinant DNA	
Radioisotopes	
Narcotics and Dangerous Drugs	
Biological Hazards	

Comments:

Evaluation Criteria

1. Significance
2. Approach
3. Innovation
4. Investigators
5. Environment

Response to Previous Reviewers (if applicable)

Overall Evaluation

Review Panel Summary (Subgrantee)

Project Lead: *DJ McMaughan*

Project Title: *Impact of social inclusion on mental wellbeing among autistic adolescents and emerging adults*

Panel:

Organization: *Oklahoma State University*

Approved: *Yes*

Reasons for Disapproval

Approved Budget

Year 1	Year 2	Year 3	Total
$43,971	*$38,724*	*$39,785*	***$122,480***

Evidence of Submission to a National Funding Organization Included (previous recipients only):

Overlapping funding

Reason for Budget Adjustment (if applicable)

Summary Comments from Review Session

The current proposal addresses mental health in autistic adolescents and young adults. This population experiences high rates of co-occurring mental health issues including self-harm. Social inclusion is posited to be a factor in mental health of autistic individuals; the proposed study aims to define and measure social inclusion and assess association with mental health. These preliminary data could be used to develop meaningful interventions and improve training with results being integrated into the Autism Foundation's training and awareness programs. The research strategy is detailed and well-designed. Sharing sessions will be used to develop a survey to measure social inclusion, including the components of interpersonal relationships and community participation; pilot testing will be following by the deployment of the survey as well as other measures. This sample of 100 autistic individuals and 100 caregivers will provide the association data. The research is highly significant, the environment is excellent, and the investigators are uniquely qualified to conduct the research. Weaknesses were minor, including a lack of clarity about exactly how caregiver data would be used, (especially for the subset of caregivers linked to a respondent) and why only one measure of well-being was included. The resubmission was responsive to prior critique.
